# A Novel Treatment With Obinutuzumab-Chlorambucil in a Patient With B-Cell Prolymphocytic Leukemia: A Case Report and Review of the Literature

**DOI:** 10.1177/2324709618788674

**Published:** 2018-07-17

**Authors:** Jason Hew, Dat Pham, Trevanne Matthews Hew, Vinay Minocha

**Affiliations:** 1 University of Florida, Jacksonville, FL, USA

**Keywords:** B-cell prolymphocytic leukemia, obinutuzumab, elderly patient with leukemia, anti-CD20 monoclonal antibody

## Abstract

We report the case of a patient with B-cell prolymphocytic leukemia who was successfully treated with the novel humanized monoclonal antibody obinutuzumab. This patient was previously treated with the combination of rituximab and bendamustine and had recurrent infusion reactions. Her treatment with rituximab and bendamustine was discontinued when she developed disease progression after 3 cycles of therapy. She was then treated with obinutuzumab 1000 mg on day 1 of every cycle and chlorambucil 0.5 mg/kg on days 1 and 15 every 28 days to which she had greater tolerability. After 4 cycles of treatment, she had resolution of her clinical symptoms, massive splenomegaly, and normalization of her white blood cell count.

## Background

B-cell prolymphocytic leukemia (B-PLL) is a rare mature B-cell neoplasm that typically occurs in the elderly population. This disease is clinically characterized by a rapidly rising lymphocyte count, splenomegaly, fever, night sweats, and weight loss. Lymphadenopathy is not generally a prominent feature of this malignancy. The diagnosis of B-PLL is made on a combination of immunophenotypic and genetic findings in the peripheral blood and bone marrow. By definition the diagnosis requires that the prolymphocytes exceed 55% of all lymphoid cells in the peripheral blood.^1^ Flow cytometry is used to distinguish B-PLL from similar neoplasms and usually demonstrates light chain restriction, bright surface immunoglobulin, and the expression of B-cell antigens including CD20, CD22, FMC7, and CD79a. CD5 and CD23 expression is often weak or absent. CD11c, CD103, CD10, and CD25 are not expressed.^1^ Tumors demonstrating t(11;14)(q13;q32) must be tested by either conventional cytogenetics, fluorescence in situ hybridization, or by immunohistochemical stains for cyclin D1 to exclude the diagnosis of mantle cell lymphoma.

B-PLL frequently follows an aggressive clinical course and has historically been associated with a poor prognosis with an estimated median overall survival of 3 years.^2^ Despite advances in the understanding of tumor biology, optimal treatment options have not yet been identified and there are no randomized studies available for clinical reference. Treatment strategies have therefore been fashioned from that of similar but more common neoplasms including chronic lymphocytic leukemia (CLL) and mantle cell lymphoma. Conventional chemotherapy has been used in the past including combination regimens such as cyclophosphamide, doxorubicin, Oncovin, and prednisolone (CHOP), which has yielded only partial responses. In addition, these responses are not durable with relapses usually occurring within 12 months. Another option for selected patients is allogeneic stem cell transplant. This offers curative potential and there are reports of long-term remissions exceeding 5 years. Allogeneic stem cell transplant, however, is best reserved for the few, younger and fit patients with this disease. It is associated with a high risk of morbidity and mortality. More recently, there have been case reports of treatments with the combination of the anti-CD20 monoclonal antibody, rituximab, and chemotherapy, which have produced excellent responses.^2^ There are, however, no known reports of the more novel humanized type II anti-CD20 monoclonal antibody obinutuzumab in the treatment of B-PLL. Obinutuzumab has been compared head to head with rituximab and has yielded superior results in the management of CLL in a pivotal phase III trial.^3^ This drug is approved by the Food Drug Administration (FDA) for first-line use in CLL and is a preferred regimen in the elderly.

## Case Report

**Figure 1.**
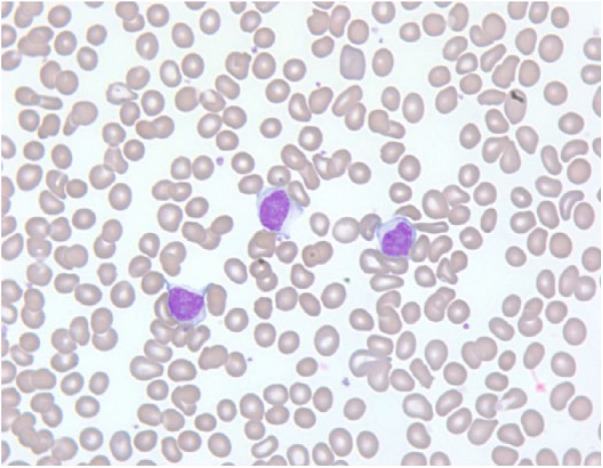
Peripheral blood smear showing 3 medium to large prolymphocytes with moderate basophilic cytoplasm, indented nuclei, and prominent vesicular nucleoli. These prolymphocytes account for >55% of the circulating cells.

**Figure 2.**
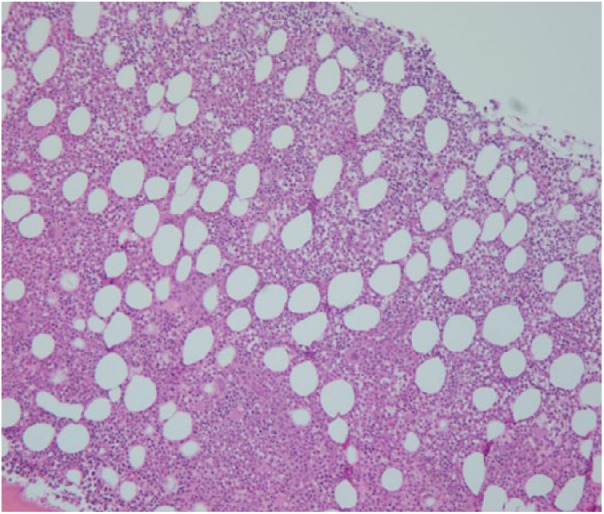
Bone marrow biopsy shows a marrow diffusely infiltrated by predominantly CD20-positive B-lymphocytes accounting for about 50% of marrow cellularity. B- and T-lymphocyte ratio is estimated to be 2:1. The infiltration is interstitial and with focal micronodularity.

**Figure 3.**
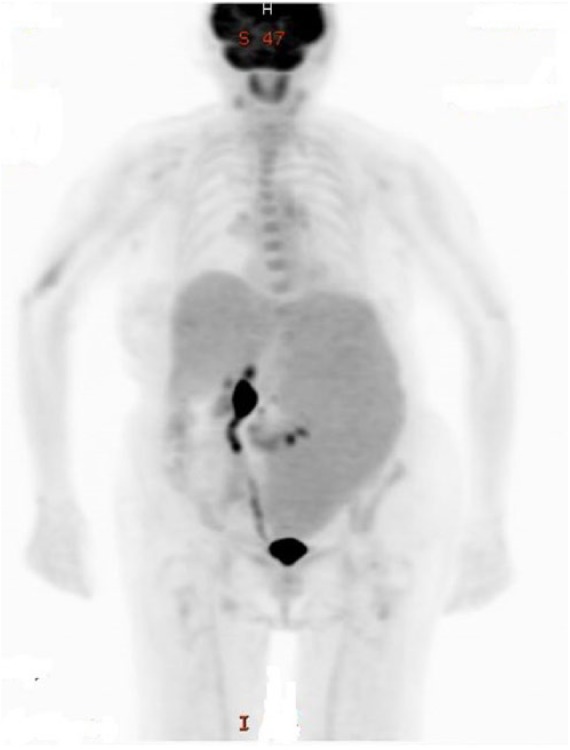
Positron emission tomography/computed tomography at the time of diagnosis and prior to the initiation of treatment. The spleen is massively enlarged, measuring 29.2 × 11.4 × 11.7 cm. There is homogeneous density and homogeneous FDG activity similar to the liver with a maximum SUV of 2.6.

A 78-year-old female was referred to our clinic for evaluation of anemia and thrombocytopenia. She complained of fatigue, early satiety, and had an unintentional weight loss of 80 pounds over the past 2 years. She denied fevers, night sweats, nausea, vomiting, or abdominal pain. Physical examination revealed massive splenomegaly, but no hepatomegaly or lymphadenopathy. A complete metabolic profile and lactate dehydrogenase were normal. Her hemoglobin and platelet counts were 10.0 g/dL and 91 × 10^9^/L, respectively. Her white blood cell count was 8.7 × 10^9^/L with 67% lymphocytes and 5% atypical lymphocytes. The peripheral smear showed abundant prolymphocytes (Figure 1). A bone marrow aspirate and biopsy revealed a marrow that was diffusely infiltrated by atypical, homogenous lymphocytes with medium to large size moderately condensed chromatin and prominent nucleoli. These lymphocytes accounted for about 50% of marrow cellularity, with B- and T-lymphocyte ratio estimated to be 2:1 (Figure 2). Flow cytometric analysis of the bone marrow aspirate with additional markers revealed that B cells were positive for CD20 and FMC7 (relatively dim and variable) and negative for CD23 and surface immunoglobulin M and immunoglobulin D. The bone marrow pathology and immunophenotype was consistent with a diagnosis of B-PLL. Cytogenetic analysis of the bone marrow aspirate revealed no chromosomal abnormalities. A positron emission tomography (PET) scan revealed the spleen to be massively enlarged measuring 29 × 12 × 8 cm with significant mass effect on the intra-abdominal contents, with displacement of the left kidney to the midline and compression of the colonic splenic flexure (Figure 3). There were also a few scattered mildly hypermetabolic lymph nodes throughout the body. Her initial ECOG (Eastern Cooperative Oncology Group) performance status was 1.

**Figure 4.**
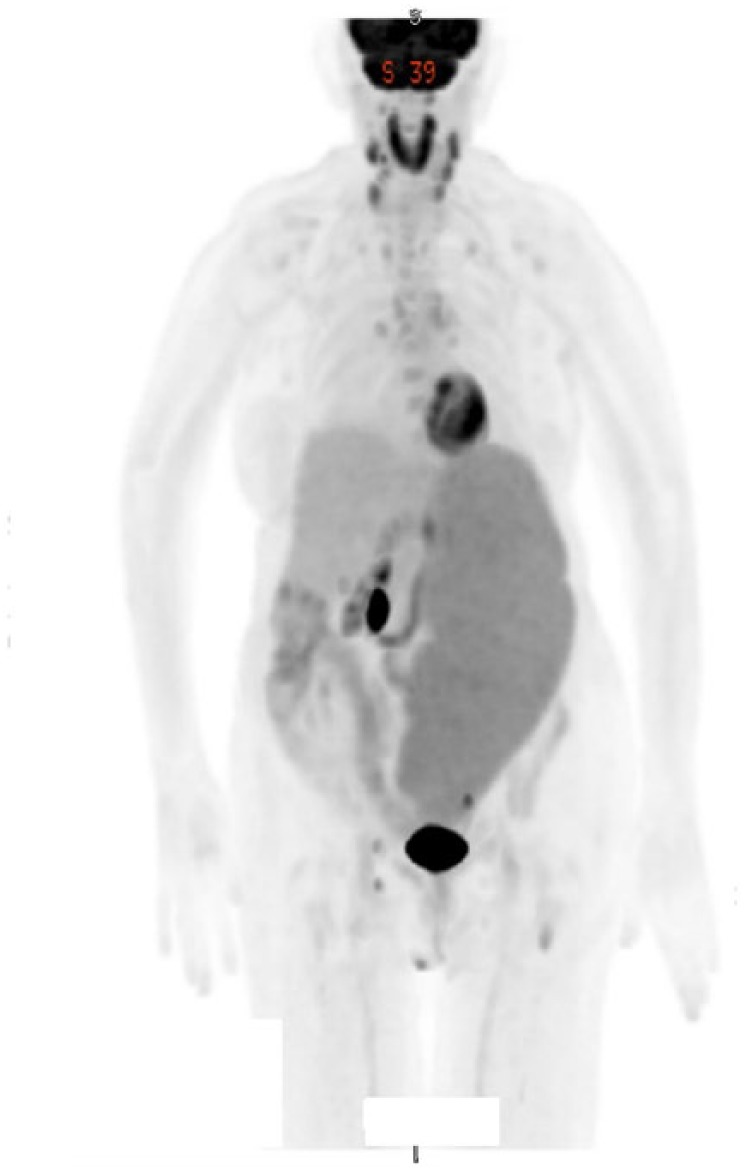
Positron emission tomography/computed tomography showing disease progression after treatment with rituximab and bendamustine. The study was done prior to her initial treatment with obinutuzumab and chlorambucil. There is interval development of bilateral cervical, mediastinal, and periportal adenopathy. The spleen is massively enlarged measuring 29 × 12 × 8 cm. There is diffuse increased uptake within the spleen with maximum SUV being 3.2.

**Figure 5.**
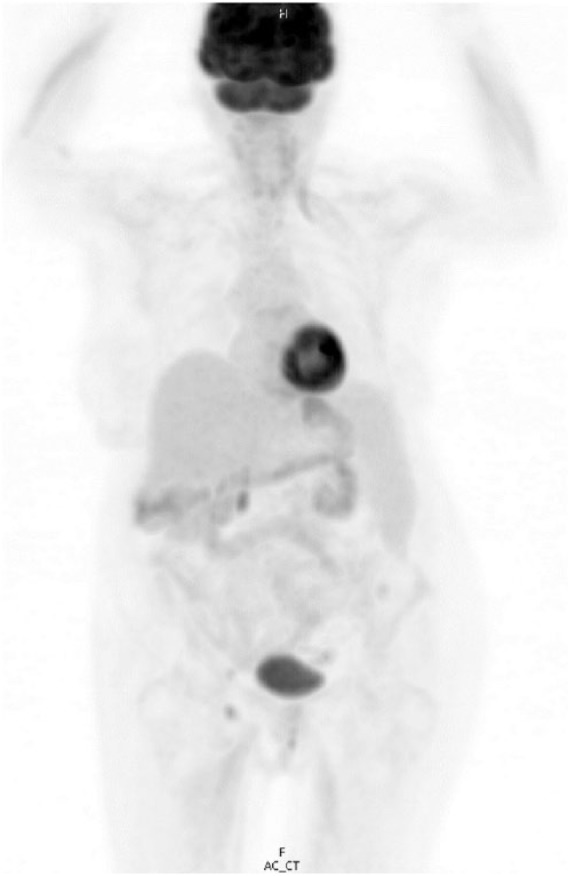
Positron emission tomography/computed tomography after 4 cycles of treatment with obinutuzumab and chlorambucil. The spleen is decreased in size compared with prior study, now measuring approximately 18.5 × 10.0 × 7.3 cm and decreased in uptake with maximum SUV now measuring 1.9, which is slightly above blood pool and less than liver uptake.

The patient was started on treatment with rituximab and bendamustine; however, she experienced recurrent infusion reactions with rituximab, which worsened with every treatment. Rituximab was stopped after the third cycle. After receiving 3 cycles of treatment, a repeat PET scan showed interval development of bilateral cervical hypermetabolic lymphadenopathy, persistent mediastinal, periportal hypermetabolic lymph nodes, and persistent splenic enlargement. She also developed bilateral pleural effusions (Figure 4). Her posttreatment hemoglobin and platelet counts were 10.0 g/dL and 50 × 10^9^/L, respectively. Her white blood cell count decreased to 5.3 × 10^9^/L but consisted predominantly of lymphocytes and prolymphocytes. She was therefore considered to have disease progression because of her persistent cytopenias, the progressive worsening of her lymphadenopathy and splenomegaly, and overall decline in her performance status to an ECOG of 2. She expressed a desire to continue treatment for her B-PLL, so it was decided to change therapy to a combination of obinutuzumab and chlorambucil. This regimen entailed the administration of obinutuzumab 1000 mg on day 1 of every cycle and chlorambucil 0.5 mg/kg on days 1 and 15 every 28 days. She demonstrated greater tolerability to this regimen and did not experience any infusion reactions. After 4 cycles of obinutuzumab-chlorambucil, she had gained weight with improved appetite and energy level. On physical examination, her spleen was no longer palpable. A repeat posttreatment PET scan showed a decreased spleen size, which measured approximately 18.5 × 10.0 × 7.3 cm, and her lymphadenopathy and pleural effusions had resolved (Figure 5). She also had an improvement in her anemia and thrombocytopenia posttreatment with normalization of the lymphocyte count. Prolymphocytes were no longer noted on the peripheral smear. She achieved a clinical partial remission and was then placed on maintenance obinutuzumab 1000 mg monotherapy every 2 months. In total she completed 8 cycles of treatment with obinutuzumab. Therapy was held thereafter as she had no evidence of active disease and she was switched to surveillance. No major adverse events occurred during her treatment with obinutuzumab and chlorambucil or while on monotherapy with obinutuzumab. At the time of this report, she was still alive at 81 years old with no evidence of disease progression and has been off treatment for greater than a year.

## Discussion

In this study, we report the case of an elderly female with a diagnosis of B-PLL who displayed an excellent clinical response to chemo-immunotherapy with a combination of obinutuzumab and chlorambucil after failing initial treatment with bendamustine and rituximab. Cases of B-PLL are extremely rare, accounting for 1% of lymphocytic leukemias,^1^ and consequently, there are very little published data regarding optimal treatment for this disease. There is a considerable overlap in the clinicopathologic presentation of this disorder and other mature B-cell leukemias/lymphomas, such as CLL, hairy cell leukemia variant, and splenic marginal zone lymphoma. Most treatment data have been derived from case reports or small series and have been extrapolated to include regimens that have been used in the more common B-cell disorders.^2,4^ Although previous cases have described good outcomes with a conventional chemo-immunotherapy approach using rituximab, we describe the first case of a patient with B-PLL who had a successful outcome using a combination of obinutuzumab and chlorambucil.

The development of monoclonal antibodies have had a major impact in altering the natural history of both malignant and benign immune-mediated diseases. Rituximab is the first FDA licensed and still the most commonly used anti-CD20 monoclonal antibody in B-cell malignancies.^5^ Since its approval for relapsed/refractory non-Hodgkin’s lymphoma in 1997, rituximab has been endorsed for use in the treatment of numerous other B-cell malignancies.^6^ This achievement ignited the era of direct monoclonal antibody therapy. The success and failure of rituximab have also improved the understanding of how monoclonal antibodies work. It has also paved the way for the development of more novel drugs that have improved efficacy. In a literature search, only a few case reports were found describing the successful treatment of B-PLL. Rituximab monotherapy and combinations of rituximab with fludarabine or bendamustine together with an anthracycline such as mitoxantrone or epirubicin (FMR, FER, and BMR) have been reported to have activity in B-PLL.^7-10^

Functionally anti-CD20 monoclonal antibodies may be classified as either type I or type II. The type I anti-CD20 monoclonal antibodies, rituximab and ofatumumab, lead to complement-dependent cytotoxicity, stimulation of signaling leading to apoptosis, and antibody-dependent cell-mediated cytotoxicity through the recruitment of immune mediator cells.^11^ Obinutuzumab is a type II anti-CD20 monoclonal antibody that recognizes the same CD20 epitope as rituximab but binds to it in a different orientation and over a larger surface area, allowing a superior induction of direct cell death, enhanced natural killer cell activation, and antibody-dependent cell cytotoxicity. It is, however, less potent in inducing complement-dependent cytotoxicity.^3^ The mechanism of action of obinutuzumab, in contrast to that of rituximab and ofatumumab, may provide greater efficacy.^11^ For this reason, obinutuzumab-based treatment may display a superior response in patients with B-PLL as was evidenced in our case.

The FDA authorized the use of obinutuzumab in combination with chlorambucil in 2013 for the elderly and frail patient with treatment-naïve CLL. Approval was granted based on the results of the phase III CLL-11 study. This 3-arm study compared the combination of obinutuzumab and chlorambucil versus rituximab and chlorambucil versus chlorambucil alone. The results of this study showed marked superiority of the obinutuzumab combination in response rates and survival compared with the rituximab-based regimen and chlorambucil alone. The median progression-free survival was 26.7 months with obinutuzumab-chlorambucil versus 16.3 months with rituximab-chlorambucil versus 11.1 months with chlorambucil alone. Safety and tolerance to treatment was another key measure shown in this study. Less patients died from an adverse event with obinutuzumab-chlorambucil (4%) than in the rituximab-chlorambucil and chlorambucil-alone groups (6% and 9%, respectively).^12^ The efficacy and safety data from this study served as the catalyst for using this regimen in this case.

The authors of this report also acknowledge that chlorambucil has proven efficacy in the treatment of CLL and other lymphoproliferative disorders. It may have therefore contributed significantly to the disease response observed in this case. In a systematic review by Lepretre et al, response rates of 31% to 72% were reported with chlorambucil monotherapy in the treatment of CLL; however, complete responses were as low as 0% to 10%.^13^ This oral alkylating agent was first approved for use in 1957 for the treatment of CLL. The use of chlorambucil has since largely fallen out of favor in preference to more novel agents, which have improved response rates. It is still indicated for use in the elderly and frail patient due to its safety and tolerance. Improved complete response rates and progression-free survival are seen when chlorambucil is combined with an anti-CD20 monoclonal antibody.

In conclusion, the combination of obinutuzumab and chlorambucil may have a promising role in the treatment of patients with B-PLL and deserves consideration either in the frontline setting or in the subset of patients who progress on rituximab-based regimens. More studies are needed to investigate the efficacy of this regimen in patients with B-PLL and the optimal length of therapy.
